# Intrapulmonary Vaccination Induces Long-lasting and Effective Pulmonary Immunity Against *Staphylococcus aureus* Pneumonia

**DOI:** 10.1093/infdis/jiab012

**Published:** 2021-01-08

**Authors:** Xin Fan, Ning Li, Meiyi Xu, Decheng Yang, Beinan Wang

**Affiliations:** 1 Key Laboratory of Pathogenic Microbiology and Immunology, Institute of Microbiology, Chinese Academy of Sciences, Beijing, China; 2 Savaid Medical School, University of Chinese Academy of Sciences, Beijing, China; 3 Division of Livestock Infectious Diseases, State Key Laboratory of Veterinary Biotechnology, Harbin Veterinary Research Institute, Chinese Academy of Agricultural Sciences, Harbin, China

**Keywords:** *Staphylococcus aureus* pneumonia, intrapulmonary immunization, lung tissue resident memory T cells, long-term protection

## Abstract

**Background:**

*Staphylococcus aureus* causes community- and hospital-acquired pneumonia linked to a high mortality rate. The emergence and rapid transmission of multidrug-resistant *S. aureus* strains has become a serious health concern, highlighting the challenges associated with the development of a vaccine to combat *S. aureus* pneumonia.

**Methods:**

This study evaluated the effects of intrapulmonary immunization on the immune response and protection against *S. aureus* lung infection in a respiratory mouse model using a subunit vaccine.

**Results:**

Compared with the intranasal immunized mice, the intrapulmonarily immunized mice had lower levels of pulmonary bacterial colonization and lethality, accompanied by alleviated lung inflammation with reduced proinflammatory cytokines and increased levels of interleukin-10 and antimicrobial peptide following intrapulmonary challenge. Optimal protection was associated with increased pulmonary antibodies and resident memory T cells. Moreover, intrapulmonary immunization provided long-lasting pulmonary protection for at least 6 months, with persistent cellular and humoral immunity in the lungs.

**Conclusions:**

Vaccine reaching the deep lung by intrapulmonary immunization plays a significant role in the induction of efficacious and long-lasting immunity against *S. aureus* in the lung parenchyma. Hence, intrapulmonary immunization can be a strategy for the development of a vaccine against *S. aureus* pneumonia.

Immunization through the intrapulmonary route with a subunit of *S. aureus* vaccine elicited tissue resident memory T cells and antigen-specific antibodies in the lungs, and provided optimal and long-term protection against *S. aureus* pneumonia.

*Staphylococcus aureus* is associated with a wide range of infections. Invasive *S. aureus* infection, including pneumonia, is a leading cause of serious illness and death worldwide. This has become apparent by the rapidly emerging antibiotic-resistant *S. aureus* strains, which have been associated with hospital- and community-acquired pneumonias [1, 2], as well as being a complication of inﬂuenza infection [3]. There is an urgent and unmet clinical need for immune-based approaches to treat these infections, with the aim to reduce the serious threat to public health. However, to date, all attempts in human trials to develop a vaccine for the prevention of *S. aureus* invasive infections have failed [4, 5]. Therefore, there is an urgent need for an effective vaccine to prevent staphylococcal infection.

Pneumonia is an infection in the lung parenchyma initiated by aspirated organisms that first colonize the nasal cavity and are subsequently channeled into the lung parenchyma [6]. Immune responses in the lungs can result in the timely and optimal immune clearance of pathogens. The majority of currently approved vaccines are delivered through the parenteral route, inducing a systemic antibody that can reach the lung parenchyma for protection against pathogens. Nevertheless, parenteral immunization induces poor immune responses at the respiratory mucosal surface, and does not protect against pathogen colonization of the upper respiratory tract [7]. Lately, the intranasal (i.n.) route targeting respiratory mucosa has become an appropriate method of immunization because it induces immunity to pathogens at both the upper respiratory tract and circulation [7, 8]. More recently, intrapulmonary immunization designed to distribute antigens into the lower respiratory tract [9] has been recognized as a strategy for the development of a pneumonia vaccine, aiming at the efficient induction of a local immune response in the lung parenchyma [10, 11]. Although induction of pulmonary immunity has been recognized as an important strategy in the development of a vaccine for some other pneumonia pathogens, it has not been investigated for *S. aureus* pneumonia.

Immune memory confers long-term protection and is the basis for efficacious vaccines. Immune memory is provided by long-lasting antibodies and T cells. Besides central memory cell and effector memory cell subsets, a third subset of memory T cells, referred to as tissue resident memory T cells (Trm), has been recognized. These cells do not recirculate in the blood, and can localize at the site of infection as a first line of defense against pathogens [12]. Their crucial roles in the enhanced host regional immunity have been considered for the generation of new and more effective vaccines to reduce the incidence of numerous infectious diseases [13–15]. It was found that Trm cells are confined to the previously infected lobe, and protection against pneumonia is limited to that immunologically experienced lobe [16]. This evidence indicates that Trm preferentially populate the site of induction/immunization [17]. It has been reported that intrapulmonary immunization induces an equivalent serum immunoglobulin G (IgG) response to that induced by an injected vaccine [18], and also long-lasting IgG and immunoglobulin A (IgA) responses in samples of both blood and bronchoalveolar lavage fluid (BALF) [10]. These findings indicate that immunization through the intrapulmonary route is more promising than other delivery routes for the establishment of protective immunity against lung infection [19]. However, pulmonary Trm have not been studied for protective immunity against *S. aureus* pneumonia.

Staphylococcal clumping factor A (ClfA) is a highly conserved fibrinogen-binding protein that contributes to tissue adhesion and initiation of infection [20]. ClfA is currently a potential target of *S. aureus* vaccines that can induce both B- and T-cell responses [21, 22]. In this study, ClfA was selected as a vaccine antigen to investigate the impact of the immunization route on the immune response and effectiveness of protection. We demonstrated that, compared with i.n. immunization, mice that received intrapulmonary immunization had lower levels of pulmonary bacterial colonization and a higher survival rate. Analyses of immune responses revealed that pulmonary antibody and Trm were efficiently induced by immunization through the intrapulmonary but not the i.n. route. In addition, intrapulmonary immunization provided persistent protection for at least 6 months, with higher levels of long-lasting interleukin 17^+^ (IL-17^+^) T cells and IgG- and IgA-producing B cells.

## MATERIALS AND METHODS

### Ethics Statement

This study was performed in strict accordance with the recommendations in the Guide for the Care and Use of Laboratory Animals of the Institute of Microbiology, Chinese Academy of Sciences (IMCAS) Ethics Committee. The protocol was approved by the Committee on the Ethics of Animal Experiments of IMCAS (permit number SQIMCAS2019058). Mice were bred under specific pathogen-free conditions in the laboratory animal facility at IMCAS. All animal experiments were conducted under isoflurane anesthesia, and all efforts were made to minimize suffering.

### Mice, Immunization, and Challenge

Female C57BL/6 (B6) mice (aged 4–6 weeks) were purchased from Vital River Laboratory Animal Technology (Beijing, China); all colonies were introduced from Charles River Laboratories (Wilmington, Massachusetts). Mice were anesthetized by intraperitoneal injection with pentobarbital sodium (60 mg/kg) and subsequently inoculated with puriﬁed ClfA_221–550_ (10 μg) [23] and cholera toxin B subunit (CTB) (1 μg) (Sigma, St Louis, Missouri). For intrapulmonary inoculation, ClfA and CTB in 30 μL of phosphate-buffered saline (PBS) were intranasally delivered to the left nostril to ensure the inoculated antigens reaching the parenchyma [24]. For i.n. inoculation, the vaccine was suspended in 10 μL of PBS, and 5 μL of the suspension was delivered to each nostril. Control mice received 1 μg of CTB in an equal volume of PBS through the i.n. or intrapulmonary route accordingly. Mice were immunized thrice with 2-week intervals. Immunized mice were intrapulmonarily challenged with *S. aureus* at 2 × 10^8^ (in 50 μL of total volume)/mouse 3 weeks after the last immunization. Bacterial numbers in the lungs were determined by counting the colony-forming units (CFUs) 24 hours following challenge. For the survival assay, mice were intrapulmonarily infected with the bacteria (4 × 10^9^/50 μL/mouse) 3 weeks after the last immunization, and weight loss and survival were monitored twice per day for 10 days. Mice with weight loss ≥25% of the starting body weight were euthanized and recorded as dead. Mortality was an anticipated outcome and approved by the animal ethics committee.

### Isolation of Pulmonary Mononuclear Cells

Mouse pulmonary mononuclear cells were isolated as described by Gibbings and Jakubzick [25] with modification. In brief, the lungs were harvested after perfusion with PBS, cut into small pieces, and digested with collagenase type IV (540 U/mL; Worthington Biochemical Corporation, Lakewood, New Jersey) in 1640 medium for 45 minutes at 37°C. Single-cell suspensions were prepared by mechanical dissociation of lung tissue through a 70-μm nylon mesh; cells were harvested, suspended in PBS, and isolated using standard density gradient techniques.

### Intravascular Staining for the Discrimination of Vascular and Lung Tissue Leukocytes and Flow Cytometry Analysis

Intravascular staining for the discrimination of vascular and lung tissue leukocytes was conducted as previously described [26]. Anti-CD45.2 PE (clone 104, catalog number 12-0454-81; eBioscience, San Diego, California) was diluted to 25 mg/mL in sterile PBS; 100 μL of the solution was intravenously injected via the mouse tail 3 minutes before sacrificing the mice. The stained CD45.2^+^ cells are blood-borne in the mouse lungs and can be discriminated from cells localized in the lung tissue. Cellular staining and flow cytometry analyses for T helper (Th) cells and neutrophils were conducted as previously described [27]. In brief, cells were stained for surface markers with anti-CD4 fluorescein isothiocyanate (FITC; clone: GK1.5; eBioscience) for Th cells, and with anti-CD11b FITC (clone: M1/70; Biolegend, San Diego, California) and anti-Gr-1 APC (clone: RB6-8C5; Biolegend) for neutrophils. For intracellular staining, fixed cells were permeabilized and stained with anti–IL-17 FITC (catalog number 11-7177-81; eBioscience) and anti–interferon gamma (IFN-γ) APC (clone: XMG1.2; eBioscience) for 60 minutes in the dark at 4°C. Cells were washed with 1 mL of permeabilization buffer and resuspended in staining buffer for analysis. Samples were analyzed using a FACSCalibur or FACSCanto flow cytometer (BD Biosciences) and the FlowJo software (Tree Star, Ashland, Oregon).

### Statistical Analysis

Each experiment was performed at least twice with 3–6 mice or samples per group. One- or 2-way analysis of variance followed by Tukey multiple comparisons test was used to analyze the difference between multiple groups. Survival curve was analyzed using the log-rank test (GraphPad Prism, version 7.0 for Windows; GraphPad Software, San Diego, California). Significance was reached at *P* < .05. Data were expressed as the mean ± standard error of the mean. 

Details regarding the bacterial strains and growth conditions, cloning and expression of recombinant ClfA_221–550_, sample collection and preparation, enzyme-linked immunosorbent assay (ELISA), histological analysis of lung tissue, and enzyme-linked immunospot assay (ELISpot) are specified in the Supplementary Materials and Methods.

## RESULTS AND DISCUSSION

### Immunization Through the Intrapulmonary Route Reduced *S. aureus* in the Lungs More Efficiently Than Immunization Through the Intranasal Route When ClfA Was Used as Antigen

**Figure 1.**
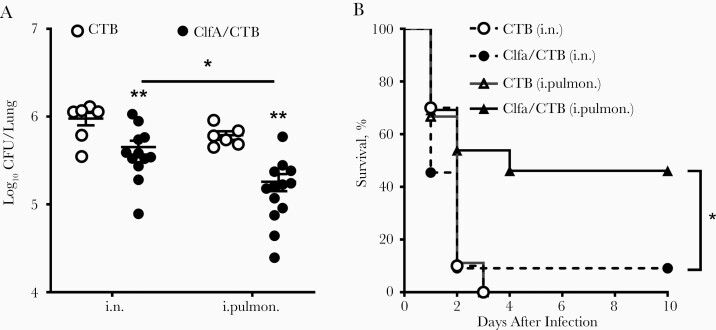
Immunization through the intrapulmonary (i.pulmon.) route provided better protection against *Staphylococcus aureus* infection. Mice were immunized through the intranasal (i.n.) or i.pulmon. route with clumping factor A (ClfA) and cholera toxin B subunit (CTB) or with CTB alone as control thrice with 2-week intervals. Three weeks after the last immunization, mice were i.pulmon. challenged with *S. aureus. A*, Colony-forming units (CFUs) in homogenized lung tissue were determined 24 hours following challenge (n = 6–8). *B*, Mice were i.pulmon. challenged with a high dose of *S. aureus* and monitored daily over 10 days (n = 10 per group). Data are presented as the mean ± standard error of the mean of 2 independent experiments. *P < .05, **P < .01; 2-way analysis of variance with Tukey posttest (*A*) or log-rank test (*B*).

The anatomical compartmentalization of immune responses is crucial in the efficacy of vaccines and imposes constraints in the selection of topical route(s) of vaccine administration. We hypothesized that the induction of immune response in the upper respiratory mucosa and the lung parenchyma would provide better protection against infection in the respiratory tract. ClfA, a proven protective *S. aureus* antigen, was used to test this hypothesis. The purity of the recombinant protein reached 95% (Supplementary Figure 1). Mice were inoculated through the left nostril with 10 μg of ClfA_221–550_ [23] and 1 μg of CTB in 30 μL of PBS. This volume allows the antigen to reach the lung tissue (intrapulmonary) [28]. One group of mice was inoculated with the same dose of the antigen in the same manner; however, the volume was reduced to 10 μL (5 μL per nostril). This low volume deposits antigen in the upper respiratory tract (i.n.) [28]. Control mice were inoculated with 1 μg of CTB in 30 μL or 10 μL of PBS through the intrapulmonary or i.n. route, respectively. Three weeks after the last immunization, the protective effect was determined by the number of CFUs in the lungs 24 hours after intrapulmonary challenge with *S. aureus*. We found that the number of CFUs was substantially lower in the i.n. group compared with the CTB control group, and further reduced in the intrapulmonary group (Figure 1A). One set of immunized mice was also challenged with a higher dose of the bacteria, and the survival rate was monitored daily over 10 days. As shown in Figure 1B, while all mice in the control group and 90% in the i.n. group expired within 3 days, 50% of mice in the intrapulmonary group survived until the end of the experiment. These results indicated that immunization through the intrapulmonary route provides more effective protection against lung infection and reduces lethality.

### Less Severe Inflammation and Pathology in the Lungs Was Found in Intrapulmonarily Immunized Than in Intranasally Immunized Mice

**Figure 2.**
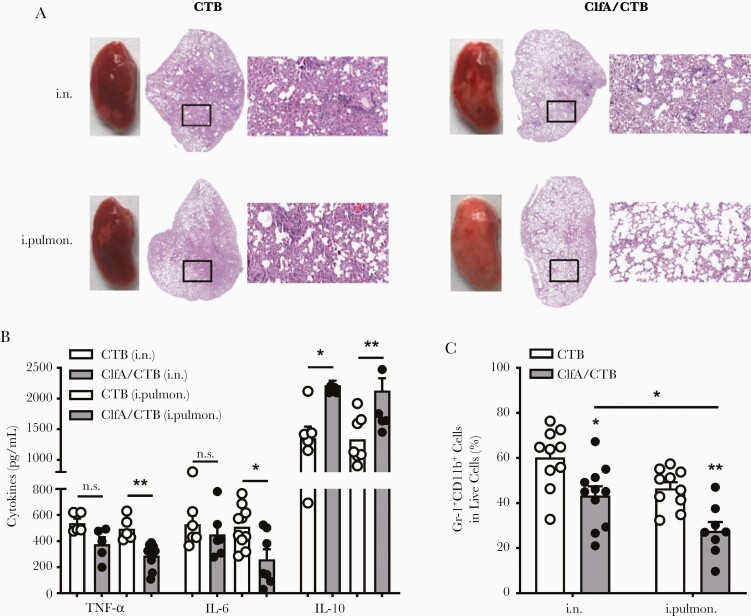
Less severe inflammation and pathology in the lungs was found in intrapulmonarily than intranasally immunized mice. Mice were immunized and challenged as described in Figure 1A. *A*, Histological stains of lung tissue specimens obtained from mice infected with *Staphylococcus aureus* 3 weeks after the last vaccination (magnification: ×20). *B*, The expression levels of tumor necrosis factor-α, interleukin 6, and interleukin 10 in the supernatants of lung homogenates were determined by enzyme-linked immunosorbent assay (n = 5–10). *C*, Lung monocytes were stained with FITC-CD11b and APC-Gr-1 and analyzed by flow cytometry (n = 8–11). Data are presented as mean ± standard error of the mean of 2 or 3 independent experiments. *P < .05, **P < .01, n.s., not significant; 2-way analysis of variance with Tukey posttest. Abbreviations: ClfA, clumping factor A; CTB, cholera toxin B subunit; IL-6, interleukin 6; IL-10, interleukin 10; i.n., intranasal; i.pulmon., intrapulmonary; TNF-α, tumor necrosis factor-α.

Inflammation triggered by infection is important for pathogen clearance and induction of adaptive responses. However, an exaggerated inflammatory response, characterized by the overproduction of proinflammatory mediators and accumulation of inflammatory cells in the lungs, can lead to intense lung injury, contributing to disease severity and mortality [29]. Therefore, a balanced inflammatory response is a key requirement for the maintenance of lung physiology and immune clearance of pathogens. A lung section was prepared to determine pulmonary pathological changes in the lung infection model 24 hours following challenge. As shown in Figure 2A, after challenge, CTB control mice showed loss of alveolar architecture, hemorrhage, infiltration of immune cells, and consolidation of the lung parenchyma. Alveolar space and less infiltration of inflammatory cells were found in the lungs of the i.n. group. However, markedly more alveolar space and clear architecture were observed in the intrapulmonary group. High levels of proinflammatory mediators and accumulation of inflammatory cells in the lungs cause tissue damage. The levels of proinflammatory cytokines were determined to confirm the observed lung pathology. ELISAs revealed that the levels of tumor necrosis factor–α and interleukin 6 in the lungs of the intrapulmonary group, but not in the i.n. group, were lower than those in the CTB control group (Figure 2B). In contrast, the level of interleukin 10, an anti-inflammatory cytokine, was increased in both the i.n. and intrapulmonary groups (Figure 2B). Flow cytometry analysis revealed that the number of neutrophils in the lungs of both the i.n. and intrapulmonary groups was notably reduced (Figure 2C). Greater reduction in the number of cells was observed following immunization of the mice through the intrapulmonary vs the i.n. route, explaining the controlled pulmonary inflammation and better maintained alveolar structure.

### Immunization Through the Intrapulmonary Route Induced Antigen-Specific Antibody Response in the Lungs

**Figure 3.**
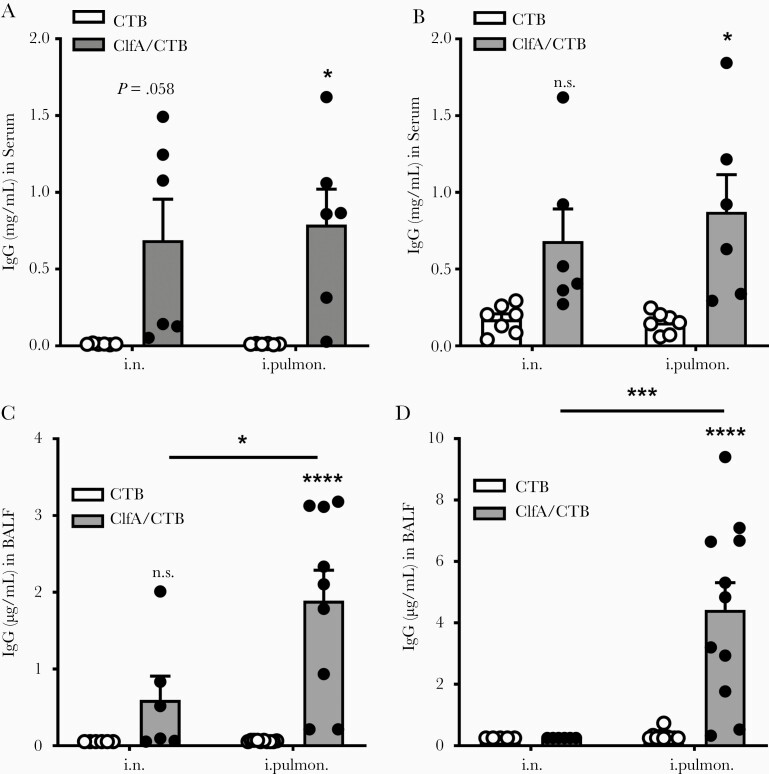
Immunization through the intrapulmonary route induced antigen-specific antibody response in the lungs. Mice were immunized and challenged as described in Figure 1A. Serum and bronchoalveolar lavage fluid (BALF) samples were collected 24 hours after challenge. The clumping factor A (Clf)–specific immunoglobulin G (IgG) (*A*) and immunoglobulin A (IgA) (*B*) in the serum, and the ClfA-specific IgG (*C*) and IgA (*D*) in the BALF were determined by enzyme-linked immunosorbent assay (n = 6–11). Data are presented as mean ± standard error of the mean of 2 independent experiments. *P < .05, ***P < .001, ****P < .0001, n.s., not significant; 2-way analysis of variance with Tukey posttest. Abbreviations: BALF, bronchoalveolar lavage fluid; ClfA, clumping factor A; CTB, cholera toxin B subunit; IgA, immunoglobulin A; IgG, immunoglobulin G; i.n. intranasal; i.pulmon., intrapulmonary.

Antibodies play an important role in facilitating antibody-mediated phagocytosis by neutrophils and monocytes/macrophages or by neutralizing virulence factors [4, 7]. Although an antibody response in the nasal passages is important in protecting against primary colonization with lung pathogens, the induction of topical antibodies in the lower respiratory tract is crucial for the efficient eradication of pathogens in the lungs and prevention of bacterial spread through the body. Pulmonary vaccination can lead to stimulation of an IgG-mediated immune protection in the alveoli and mucosal secretory IgA-mediated immune protection in the airways [10]. We hypothesized that, besides a systemic antibody response, intrapulmonary immunization could also induce antibodies in the lower respiratory tract. ELISAs revealed that the levels of ClfA-specific serum IgG and IgA were substantially elevated in the intrapulmonary group 2 weeks following the last immunization (Figure 3A and 3B). Secretory IgA (SIgA) is a key component of the first line of defense, and dominantly induced at mucosal sites by mucosa-associated lymphoid tissue, including bronchus-associated lymphoid tissue (BALT). BALT can be found beneath the epithelium of the upper bronchi, as well as in the lower airways of the lungs [30]. It is involved in the immune response and produces SIgA that can inhibit inflammation [31] and promote immune clearance [32, 33]. BALF was collected to determine the antibody response in the lower respiratory tract. We found that, unlike in serum samples, antibodies in BALF (especially SIgA) were induced in the ClfA/intrapulmonary group but not the ClfA/i.n. group (Figure 3C and 3D).

There are 2 major sources of antibodies in lung secretions, represented in the blood plasma derived from the vascular compartment by transudation across the lung tissue, and the local production by BALT of the upper bronchi and lower airways of the lungs [34]. Interestingly, we did not find increased IgG and IgA levels in BALF from the ClfA/i.n. group, although these antibodies were elevated in the blood of these mice. There is currently no explanation for this observation. Nevertheless, the results indicated that deposition of antigens into the lower respiratory tract through the intrapulmonary route can activate B cells in BALT in the lungs. The association of elevated SIgA in BALF with lower CFUs in the lungs indicated that the presence of a high concentration of antibodies in the lungs contributes to the alleviated lung inflammation and more efficient eradication of bacteria.

### Immunization Through the Intrapulmonary Route Induced Pulmonary Th17 Response and Lung Resident Memory T Cells

**Figure 4.**
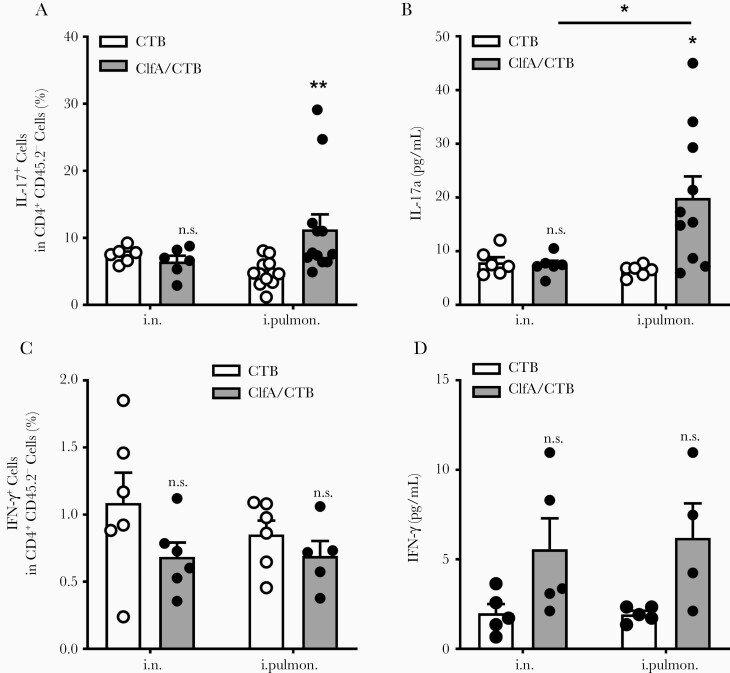
Immunization with clumping factor A through the intrapulmonary route induced pulmonary Th17 response. Six days after challenge, T cells in the lungs were determined by flow cytometry. *A*, Lung parenchymal Th17 cells (CD4^+^ IL-17^+^ CD45.2^−^) and (*C*) Th1 cells (CD4^+^ IFN-γ ^+^ CD45.2^−^) (n = 5–12). The expression of IL-17a (*B*) and IFN-γ (*D*) in the supernatants of lung homogenates was determined by enzyme-linked immunosorbent assay (n = 4–10). Data are presented as mean ± standard error of the mean of 2 or 3 independent experiments. *P < .05, **P < .01, n.s., not significant; 2-way analysis of variance with Tukey posttest. Abbreviations: ClfA, clumping factor A; CTB, cholera toxin B subunit; IFN-γ, interferon gamma; IL-17, interleukin 17; i.n. intranasal; i.pulmon., intrapulmonary.

Generation of Th17 cells (CD4^+^ IL-17^+^) has been recognized as a requirement for optimum protection against *S. aureus* infection in staphylococcal vaccine studies, including ClfA [35–37]. In addition, it has been demonstrated that SIgA response in the lungs is dependent on Th17 cells [38]. Flow cytometry analysis revealed that the percentage of Th17 cells in the lungs was not altered in mice receiving ClfA through the i.n. route compared with CTB/i.n. control mice; however, it was increased considerably in those receiving ClfA through the intrapulmonary route (Figure 4A). Consistently, ELISAs revealed that the levels of IL-17a in lung tissue were elevated in the intrapulmonary group but not the i.n. group (Figure 4B). Th1 cells also contribute to protective immunity against *S. aureus* infection [39, 40]. They are preferentially induced by parenteral immunization [41]. We found that the percentage of Th1 cells was not increased in mice immunized with ClfA either through the intrapulmonary or i.n route (Figure 4C). Higher levels of IFN-γ were observed in the supernatant of lung tissue obtained from these 2 groups; however, the difference was not statistically significant (Figure 4D). The cellular source of the IFN-γ may be other immune cell types, such as natural killer [42] and γδ T cells [43].

**Figure 5.**
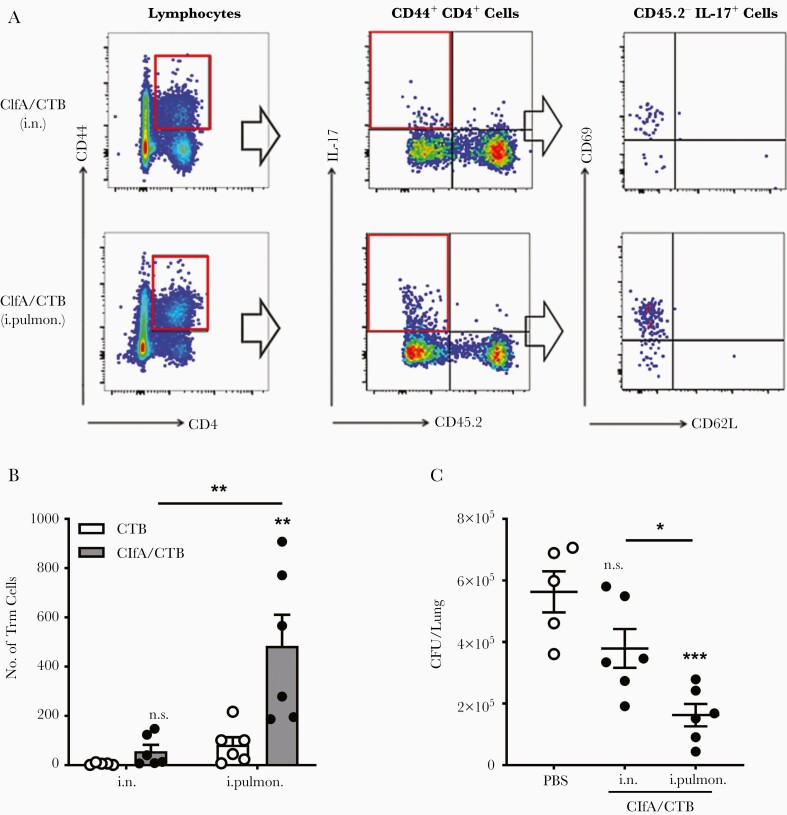
Lung resident memory T cells were induced by immunization through the intrapulmonary route. Mice were intravascularly stained by intravenous injection of phycoerythrin-conjugated CD45.2, 3 minutes prior to being sacrificed. *A*, CD44^hi^ CD4^+^ T-cell populations were subdivided according to their CD45.2 staining and expression of IL-17. CD45.2^−^ IL-17^+^ CD44^hi^ CD4^+^ T cells were further subdivided according to their CD62L vs CD69 expression. *B*, The numbers of resident memory T cells in the lung parenchyma (CD4^+^ CD44^hi^ CD69^+^ and CD62L^−^) were determined by flow cytometry and are summarized in the bar graph (n = 5–6). *C*, Mice were immunized via the intranasal or intrapulmonary route as in Figure 1A, and challenged 42 days after the last immunization. Lung tissues were harvested and homogenized for colony-forming units counting 24 hours postinfection (n = 5–6). Data are presented as mean ± standard error of the mean of 2 or 3 independent experiments. *P < .05, **P < .01, ***P < .001, n.s., not significant; 2-way analysis of variance with Tukey posttest (*B*) or 1-way analysis of variance with Tukey posttest (*C*). Abbreviations: CFU, colony-forming unit; ClfA, clumping factor A; CTB, cholera toxin B subunit; IL-17, interleukin 17; i.n. intranasal; i.pulmon., intrapulmonary; PBS, phosphate-buffered saline; Trm, resident memory T cells.

Th17 cells in the lung parenchyma can transform into Trm [38], which are persistent in the lungs and can induce a rapid and robust response to a new infection at the primary infection site, conferring protection [13, 44]. It has been reported that antigen-specific CD4^+^ Trm in the lungs play a critical role in adaptive immunity against lung infection by *Bordetella pertussis* [12] or *Mycobacterium tuberculosis* [45]. We postulated that the more effective protection observed in the intrapulmonary group was linked to pulmonary Trm. Pulmonary Trm were examined 42 days after the last immunization by flow cytometry. The analysis revealed that Trm cells (CD44^hi^ CD69^+^ CD62L^−^) were hardly found in the matching CTB control groups and not increased in ClfA/i.n. mice vs CTB/i.n. control mice (Supplementary Figure 2). However, they were substantially increased in the ClfA/intrapulmonary group (Figure 5A and 5B). To determine the link between Trm and protection efficacy, a different set of immunized mice were intrapulmonarily challenged 42 days after the last immunization, and CFUs from the lungs of these mice were determined 24 hours later. We found that the number of CFUs was lower in ClfA/i.n. mice than in CTB/i.n. control mice; however, the difference was not statistically significant. Nevertheless, substantially greater reduction in CFUs was observed in the ClfA/intrapulmonary group than the CTB/intrapulmonary group (Figure 5C). The correlation between the increased pulmonary Trm and more efficient bacterial clearance in the lungs suggests that, similar to the role of Trm in lower respiratory tract infections by other pathogens, parenchyma Trm provide superior local tissue protection against *S. aureus* in the lungs.

### Long-term Immunity Against *S. aureus* Induced by Immunization Through the Intrapulmonary Route

**Figure 6.**
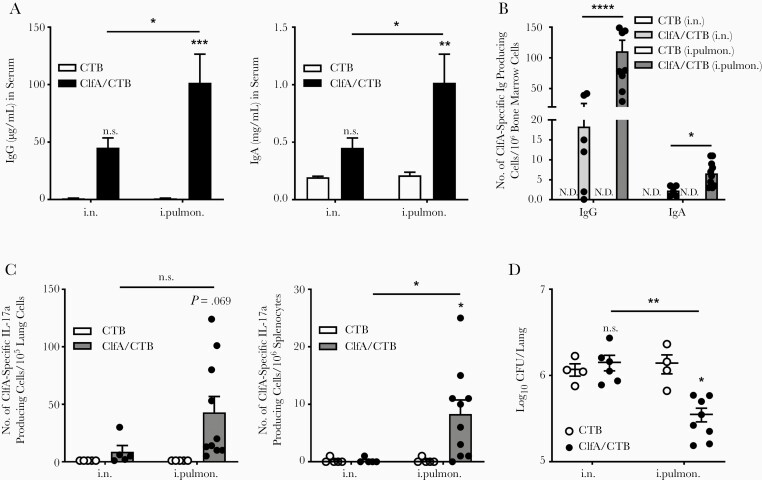
Long-term immunity against *Staphylococcus aureus* was induced by immunization through the intrapulmonary route. *A*, At 180 days after the last immunization, clumping factor A (ClfA)–specific immunoglobulin G (left) and immunoglobulin A (right) in serum samples were determined by enzyme-linked immunosorbent assay (n = 6–10). *B*, ClfA-specific long-lived plasma cells in the bone marrow were determined by enzyme-linked immunospot assay (ELISpot) (n = 5–10). *C*, ClfA-specific IL-17a–secreting cells in the lungs (left) and spleens (right) were measured by ELISpot (n = 5–10). *D*, Colony-forming units in the lungs were determined 24 hours after challenge (n = 4–8). Data are presented as mean ± standard error of the mean of 2 or 3 independent experiments. *P < .05, **P < .01, ***P < .001, ****P < .0001, n.s., not significant; 2-way analysis of variance with Tukey posttest. Abbreviations: CFU, colony-forming unit; ClfA, clumping factor A; CTB, cholera toxin B subunit; IgA, immunoglobulin A; IgG, immunoglobulin G; i.n. intranasal; i.pulmon., intrapulmonary; N.D., not detected.

The ability of the immune system to maintain a memory of previous antigens is the basis for the development of vaccines. Long-term immunological memory is provided by long-lasting antibodies and memory T cells. ELISAs were performed to determine the presence of ClfA-specific antibodies 180 days after the last immunization. The results revealed that, compared with controls, higher levels of serum IgG and IgA were maintained in mice that received immunization with ClfA through the intrapulmonary but not the i.n. route (Figure 6A). Serum antibodies are sustained by long-lived plasma cells (LLPCs) derived mainly from memory B cells primarily located in the bone marrow [46]. ELISpot assays showed higher levels of ClfA-specific IgG- and IgA-secreting LLPCs in mice immunized with ClfA through the intrapulmonary vs the i.n. route (Figure 6B). Long-lived IL-17a–secreting T cells in the lungs and spleens were also examined. T-cell ELISpots showed that the number of these cells was increased only in mice that received ClfA through the intrapulmonary route (Figure 6C). Taken together, these results indicate that intrapulmonary immunization induces long-lasting humoral and cellular immune responses. Mice were challenged 180 days following the last immunization to test the long-term protective immunity against lung infection by *S. aureus*. CFUs recovered from the lungs 24 hours following challenge showed that CFUs were substantially reduced in the ClfA/intrapulmonary group compared with the matching CTB control group. However, the number of CFUs in the ClfA/i.n. group and CTB/i.n. group was similar (Figure 6D), indicating that long-term protective immunity against *S. aureus* is established in the lungs through the intrapulmonary route.

Our results indicate that a vaccine reaching the deep lung by intrapulmonary immunization is crucial for the induction of efficacious and long-lasting immunity against *S. aureus* in the lung parenchyma. Hence, intrapulmonary immunization can be a strategy for the development of a vaccine against *S. aureus* pneumonia. Since *S. aureus* is the leading cause of skin and soft tissue infections, our findings also suggest that a better understanding of the topical maintenance of Trm is important for the development of vaccines against *S. aureus* designed to promote skin immunity.

## Supplementary Data

Supplementary materials are available at *The Journal of Infectious Diseases* online. Consisting of data provided by the authors to benefit the reader, the posted materials are not copyedited and are the sole responsibility of the authors, so questions or comments should be addressed to the corresponding author.

jiab012_suppl_Supplementary_Materials_and_Methods

jiab012_suppl_Supplementary_Figure_1

jiab012_suppl_Supplementary_Figure_2

## Notes

***Acknowledgments.*** We thank Ya Zhou, Xinxin Xing, Xingye Wang, and Shuai Bi for technical support; Dr Adong Shen (Beijing Pediatric Research Institute, Beijing Children’s Hospital, National Center for Children’s Health, Beijing, China) for sharing the *S. aureus* strain; and Junfeng Hao (Institute of Biophysics, Chinese Academy of Sciences, Beijing, China) for conducting the histological analysis of lung tissues.

***Financial support.*** This work was supported by the National Natural Science Foundation of China, China (NSFC) (31400760) to X. F. for Young Scholars and (31872743) to B. W.; and the Open Project Fund of the State Key Laboratory of Veterinary Biotechnology (award number SKLVBF2018XX to X. F.).

***Potential conflicts of interest.*** All authors: No reported conflicts of interest.
