# Supplementary material for: Intrapulmonary Vaccination Induces Long-lasting and Effective Pulmonary Immunity Against Staphylococcus aureus Pneumonia
Source: J Infect Dis. 2021 Jan 8;224(5):903–13. doi: 10.1093/infdis/jiab012 (PMC8408773; doi:10.1093/infdis/jiab012)
Supplement: jiab012_suppl_Supplementary_Materials_and_Methods [file jiab012_suppl_supplementary_materials_and_methods.docx]

**Supplementary Materials and Methods**

**Bacterial Strains and Culture Condition**

*Staphylococcus aureus* (*S. aureus*) strain 238, a clinical isolate from a patient with sepsis that was provided by the Beijing Pediatric Research Institute, Beijing Children’s Hospital (Beijing, China). The bacteria were cultured in Tryptic Soy Broth (BD bioscience, San Jose, CA, USA) for 15 h (OD_600_ ~ 1.4), harvested by centrifugation, and washed with sterile phosphate-buffered saline. Washed bacteria were diluted with phosphate-buffered saline to appropriate cell concentrations. Colony forming units of *S. aureus* were quantified on Tryptic Soy Agar (BD Diagnosis System) plates incubated overnight at 37°C.

**Cloning and Expression of Recombinant Clumping Factor A**

Genomic DNA was obtained from the *S. aureus* strain 238 using the TIANamp Bacteria DNA Kit (TIANGEN, China). The clumping factor A (ClfA) (amino acids 221–550) gene was amplified by polymerase chain reaction (PCR) using the following PCR primers and standard PCR amplification conditions: forward, 5’-TCTTATGGTGTGCTAGACATTGGC-3’ and reverse, 5’-AGGTTCTGAAACATCTGGACTTGC-3’ [1]. Recombinant ClfA was cloned into the pET28a (+) vector. The recombinant proteins were expressed in *Escherichia coli* BL21 Star (DE3) cells and purified by immobilized metal ion affinity chromatography. Bacterial lipopolysaccharide was removed following purification (≤ 0.1 EU/μg) (Solution Endotoxin Erasol; TIANDZ, Beijing China)

**Sample Collection and Preparation**

Mice were euthanized by exposure to carbon dioxide gas in a rising concentration. The lungs were removed, placed in 1 ml of pre-chilled PBS and used for the preparation of lung tissue and histological evaluation. Lung tissue was pestled and homogenized in 1 ml of PBS, and passed through a cell strainer (BD Falcon, USA) to obtain a single-cell suspension. The preparation was divided into two parts. One part was used to evaluate the CFUs, while the other was centrifuged and the supernatant was collected to measure the levels of cytokines by enzyme-linked immunosorbent assay (ELISA). The cell pellets were resuspended for flow cytometry analysis. BALF was collected as previously described [2]. Briefly, mice were euthanized and the trachea was cannulated. The lungs were secured to a blunted 8-gauge needle via the trachea and inflated with 500 μl of ice-cold PBS; subsequently, the liquid was withdrawn and collected. The process was repeated until the lungs had been lavaged with a total volume of 3 ml of PBS. The prepared BALF was stored at −80°C until analysis. Blood samples were collected from the tail veins of mice and stored at −20°C until analysis by ELISA. Samples of bone marrow and spleen cells were collected as previously described [3]. Briefly, mice were sacrificed; subsequently, the spleen and bone marrow (BM) were removed, dissociated into single-cell suspensions in complete RPMI-1640 medium, and treated with ammonium–chloride–potassium lysis buffer to lyse the red blood cells. The preparation was centrifuged, and the pelleted cells were analyzed by enzyme-linked immune absorbent spot (ELISpot) assays for antibody-producing B cells and IL-17a-producing T cells according to previously described protocols [4].

**Enzyme-linked Immunosorbent** **Assay for Cytokines and Antibodies**

Single-cell suspensions obtained from the lungs were centrifuged at 1,500 rpm for 5 min at 4°C, and the levels of tumor necrosis factor-α (TNF-α), interleukin-6 (IL-6), IL-10, IL-17a, and interferon-γ (IFN-γ) were measured using ELISA kits (eBioscience, San Diego, CA, USA) according to the instructions provided by the manufacturer and performed as previously described [5]. ClfA-specific immunoglobulin G (IgG) and IgA were detected as previously described [1]. Briefly, serially diluted sera (to 1:1,000) or undiluted bronchoalveolar lavage fluid samples were dispensed on Corning Costar 96-well plates (Thermo Fisher Scientific, Waltham, MA, USA) coated with ClfA (0.3 μg/well). Antibody binding was detected by horseradish peroxidase-conjugated anti-mouse IgG or IgA (Southern Biotech，Birmingham, AL, USA), followed by 3, 3’, 5, 5’-Tetramethylbenzidine staining (Sigma). Absorbance was measured at 450 nm (minus 630 nm for wavelength correction). Results are expressed as OD_450_ values.

**Histological Analysis of Lung Tissue**

The lungs were removed and fixed with 4% paraformaldehyde. After embedding the tissue in paraffin, sections were stained with hematoxylin-eosin according to the standard protocol and examined using a Leica SCN400 device (Germany).

**Enzyme-linked Immunospot Assays**

ELISpot assays of T and B cells were conducted as previously described [5]. Long-lived ClfA-specific IL-17a-producing T cells were examined by IL-17a ELISpot Ready-SET-Go! (catalog no. 88-7876; eBioscience, San Diego, CA, USA). ClfA-specific antibody-secreting cells were examined in MultiScreen-IP 96-well ﬁlter plates (Merck Millipore, Darmstadt, Germany) according to the instructions provided by the manufacturer. Briefly, single-cell suspensions of the lungs, bone marrow, or spleen cells (2 × 10^5^) were seeded in 96-well plates. Cells were cultured with or without ClfA (20 μg/ml) for 24 h, stained, and developed. Spots were enumerated with an ImmunoSpot Analyzer (Cellular Technology Ltd., Beachwood, OH, USA).

**References**

1. Hawkins J, Kodali S, Matsuka YV, et al. A recombinant clumping factor A-containing vaccine induces functional antibodies to Staphylococcus aureus that are not observed after natural exposure. Clin Vaccine Immunol **2012**; 19:1641-50.

2. Smith NM, Wasserman GA, Coleman FT, et al. Regionally compartmentalized resident memory T cells mediate naturally acquired protection against pneumococcal pneumonia. Mucosal Immunol **2018**; 11:220-35.

3. Zhou Y, Li S, Bi S, et al. Long-lasting protective immunity against H7N9 infection is induced by intramuscular or CpG-adjuvanted intranasal immunization with the split H7N9 vaccine. Int Immunopharmacol **2020**; 78:106013.

4. Bi S, Xu M, Zhou Y, Xing X, Shen A, Wang B. A Multicomponent Vaccine Provides Immunity against Local and Systemic Infections by Group A Streptococcus across Serotypes. mBio **2019**; 10.

5. Xing X, Bi S, Fan X, Jin M, Liu W, Wang B. Intranasal Vaccination With Multiple Virulence Factors Promotes Mucosal Clearance of Streptococcus suis Across Serotypes and Protects Against Meningitis in Mice. J Infect Dis **2019**; 220:1679-87.
