# Supplementary material for: Intrapulmonary Vaccination Induces Long-lasting and Effective Pulmonary Immunity Against Staphylococcus aureus Pneumonia
Source: J Infect Dis. 2021 Jan 8;224(5):903–13. doi: 10.1093/infdis/jiab012 (PMC8408773; doi:10.1093/infdis/jiab012)
Supplement: jiab012_suppl_Supplementary_Figure_1 [file jiab012_suppl_supplementary_figure_1.docx]

**
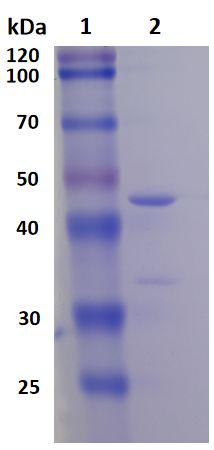
**

**Figure S1. Purified recombinant protein ClfA were analyzed by SDS-PAGE.** Lane 1, molecular weight marker; lane 2, ClfA. Abbreviations: ClfA, clumping factor A; SDS-PAGE, sodium dodecyl sulfate-polyacrylamide gel electrophoresis.
