# Supplementary material for: Intrapulmonary Vaccination Induces Long-lasting and Effective Pulmonary Immunity Against Staphylococcus aureus Pneumonia
Source: J Infect Dis. 2021 Jan 8;224(5):903–13. doi: 10.1093/infdis/jiab012 (PMC8408773; doi:10.1093/infdis/jiab012)
Supplement: jiab012_suppl_Supplementary_Figure_2 [file jiab012_suppl_supplementary_figure_2.docx]

**
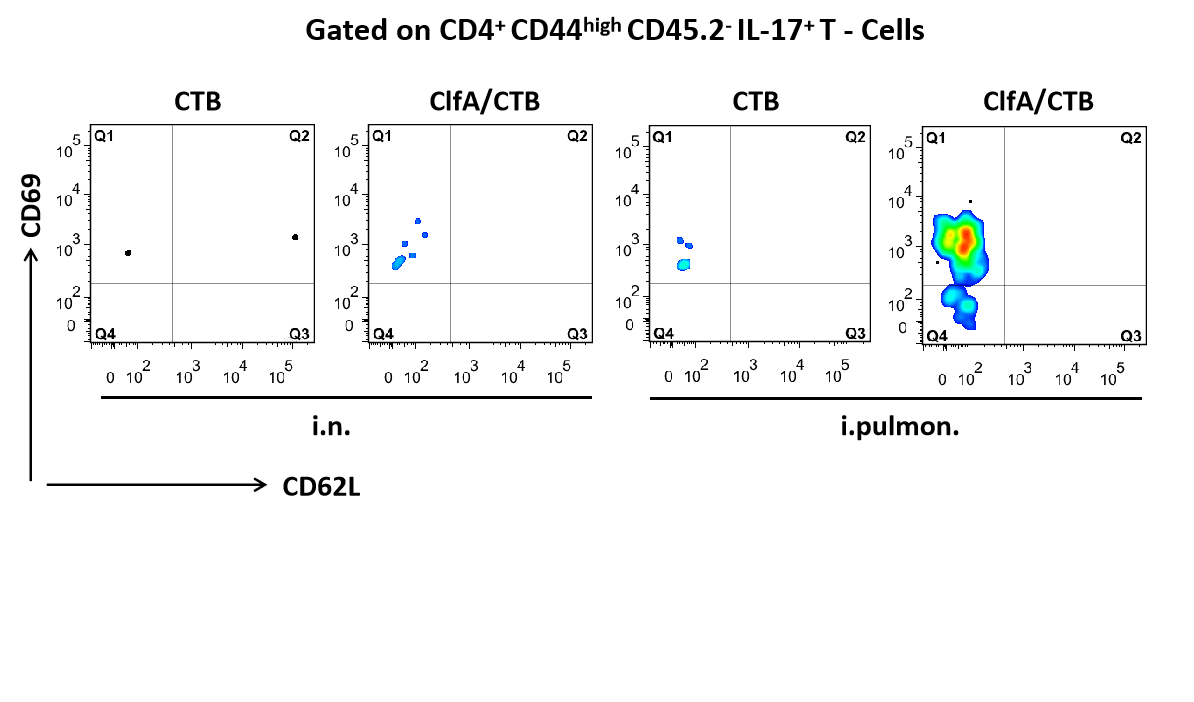
**

**Figure S2.** **Trm cells were hardly found in CTB control groups.** At 42 days after the last immunization, anti-CD45.2 mAb was intravenously injected via the tail veins of mice 3 min before the mice were euthanized. Trm in the lungs were identified by flow cytometry and examined for CD4^+^ CD44^hi^ CD45.2^−^ IL-17^+^ CD69^+^, and CD62L^−^ T cells. A representative of six mice from two experiments. Abbreviations: IL-17, interleukin-17; mAb, monoclonal antibody; Trm, tissue resident memory T cells.
